# Deep Learning Approaches for Automatic Localization in Medical Images

**DOI:** 10.1155/2022/6347307

**Published:** 2022-06-29

**Authors:** H. Alaskar, A. Hussain, B. Almaslukh, T. Vaiyapuri, Z. Sbai, Arun Kumar Dubey

**Affiliations:** ^1^Department of Computer Science, College of Computer Engineering and Sciences, Prince Sattam Bin Abdulaziz University, Al Kharj, Saudi Arabia; ^2^Department of Electrical Engineering, University of Sharjah, Sharjah P.O. Box 27272, UAE; ^3^Department of Computer Science, Liverpool John Moores University, Liverpool L3 3AF, UK; ^4^Bharati Vidyapeeth College of Engineering, New Delhi, India

## Abstract

Recent revolutionary advances in deep learning (DL) have fueled several breakthrough achievements in various complicated computer vision tasks. The remarkable successes and achievements started in 2012 when deep learning neural networks (DNNs) outperformed the shallow machine learning models on a number of significant benchmarks. Significant advances were made in computer vision by conducting very complex image interpretation tasks with outstanding accuracy. These achievements have shown great promise in a wide variety of fields, especially in medical image analysis by creating opportunities to diagnose and treat diseases earlier. In recent years, the application of the DNN for object localization has gained the attention of researchers due to its success over conventional methods, especially in object localization. As this has become a very broad and rapidly growing field, this study presents a short review of DNN implementation for medical images and validates its efficacy on benchmarks. This study presents the first review that focuses on object localization using the DNN in medical images. The key aim of this study was to summarize the recent studies based on the DNN for medical image localization and to highlight the research gaps that can provide worthwhile ideas to shape future research related to object localization tasks. It starts with an overview on the importance of medical image analysis and existing technology in this space. The discussion then proceeds to the dominant DNN utilized in the current literature. Finally, we conclude by discussing the challenges associated with the application of the DNN for medical image localization which can drive further studies in identifying potential future developments in the relevant field of study.

## 1. Introduction

In recent decades, the usefulness of medical imaging has increased the understanding and analyzing symptoms of diseases. Medical imaging techniques include X-ray, computed tomography (CT), and magnetic resonance imaging (MRI) [1]. These technologies have been used for different purposes based on the organ that is suspected for imaging and diagnosis. In the clinical setting, analysis of the medical image was usually conducted by trained experts such as radiologists and physicians in order to diagnose and understand the disease. However, these experts usually faced fatigue owing to pathologic variations and because this type of analysis requires laborious and tedious work [2]. In this sense, automated image analysis tools play an essential role in supporting clinicians to improve their examinations. Since the 1960s, researchers and doctors have attempted to take the advantage of computer-aided interventions [3]. Several tasks such as classification, segmentation, and localization are involved in the process of medical image analysis. Nevertheless, object localization (or detection) is considered a prerequisite of all previous tasks involved in medical image analysis. The key task of localization involves the detection of a specific region of interest (ROI) in a medical image, e.g., kidneys in MRI scans. All tasks of interpretation, such as the extraction of features, object recognition, classification, and segmentation, largely depend on the quality of localization. Therefore, accurate localization has become a critical step for medical image analysis. Consequently, designing robust and quick object localization methods will beneficially support the therapeutic process with regard to diagnosis, stratification of patients, treatment preparation, intervention, and follow-up [4].

Recently, numerous machine learning algorithms have been utilized for the task of localization leveraging the available medical image repositories. However, traditional machine learning methods depend on handcrafted low-level features that cannot effectively and professionally represent images. Various techniques and approaches have been developed in recent years to combat these difficulties. Lately, the explosive growth of data and the increased computing capacity has led to the design of more appropriate tools such as DL tools. Accordingly, the literature on medical imaging gained great attention for exploring the DNN in approximately 2012. Sahiner et al. [5] noted that studies using DL for radiological images increased significantly between 2016 (∼100) and 2017 (∼300). DL models provide more effective representations of complex high-dimensional datasets through their high-level professional information processing.

This study aims to present a comprehensive review of recent implementations proposed to train a DNN for ROI localization in medical images acquired from different modalities such as CT, MRI, and X-ray. As far as we are aware, this study presents the first review on object localization using DNN approaches for medical images. Besides, the study has twofold purposes. First, it orients the reader with the required background knowledge of the study, especially regarding the challenges related to medical image analysis. Second, it evaluates critically the existing literature on applications of DL approaches for medical image localization and provides possible recommendations for further developments.

The rest of this study is structured as follows: Section 2 presents the most popular DNNs developed for localization tasks. In Section 3, we highlight the major challenges that occur in medical image analysis. In Section 4, the methodology of the research is represented. In Section 5, the key content of the study which includes the evaluation of the reviewed literature is presented. Section 6 enlists the recommendations for resolving the identified challenges in enhancing the existing DNN approaches. In the end, the concluding remarks of the study with possible future research directions are discussed.

## 2. Motivation and Contribution

With the rapid advancement of the DNN, numerous survey works on medical image analysis are published in the literature. But to our surprise and knowledge, no study focused solely on examining the publications that utilize the DNN in the field of medical image localization. Motivated by this fact, the prime impetus of our study was not only to present a literature review in the domain of medical image localization based on DNN approaches but to emphasize research ideas for a new researcher by highlighting the gaps in the existing literature. Unlike most of the reviews on medical image analysis which are targeted at specific modalities or specific organ applications, our work will summarize all previous DNN works for medical image locations under one roof for aspiring researchers. As a result of this extensive research approach, we expect our work to offer researchers a one-stop point for analyzing the latest developments in the DNN as well as the research efforts focused on localizing the ROI in medical images. Furthermore, the following are the technical contributions of our work:

- Provides a systematic review of recent developments of DL approaches for localizing ROI in medical images acquired from different imaging modalities such as CT, MRI, and US
- Presents the background knowledge on dominant DNN approaches to nonexperts in the medical community
- Conveys the noteworthy results from reviewed studies in a precise and easy to understand way emphasizing certain aspects such as used experimental data, the proposed DNN architecture for localization, and the achieved research outcomes
- Sheds light and explores the challenging aspects of existing DL advances for the medical image localization process
- Outlines future directions that can inform researchers of possible prospects for research and development of effective DL models in the area of research interest

## 3. Deep Neural Networks (DNNs)

Deep learning has had remarkable success in a range of application fields in recent years. Machine learning is a fast-developing topic that has been applied to a wide range of conventional and new application fields. Various approaches have been developed based on various learning categories, such as supervised, semisupervised, and unsupervised learning. DL is a category of machine learning developed either using hierarchical learning approaches or deep architectures of learning. Learning is the process of estimating model parameters for the learned model to accomplish a certain task. In artificial neural network (ANN), for instance, the weight matrices are the parameters to be estimated. On the contrary, DL is designed with multiple layers between input and output layers. This hierarchical structure design enables to perform nonlinear information processing in multiple stages which can be used for pattern classification and feature learning. Here, representation learning is a learning method that is based on data representation and it is stated in recent work that DL-based representation learning uses hierarchical structures in which lower-level concepts are defined in terms of high-level concepts and vice versa. In some research works, DL is termed a universal learning technique owing to its potential to resolve a wide range of issues in a wide variety of contexts. To put it another way, DL is not task-specific. In the literature, there are several types of the DNN including the convolutional neural network (CNN) and long short-term memory (LSTM). The most successful and promising classes of DL architecture that have been proposed in the literature for medical image localization are discussed in this section as follows.

### 3.1. Convolutional Neural Network (CNN)

The CNN is one of the most widely utilized DL models in the field of computer vision, especially for image classification owing to its superior accuracy in comparison to other ML models. Despite the CNN outperforming other DL models, it demands more processing power and memory. Therein, the training process of the CNN is carried out on centralized high-performance systems. Since Krizhevsky et al. [6] won the ImageNet competition in 2012, CNNs have acquired tremendous popularity as an effective approach for image categorization in several disciplines. The CNN's main advantages are that it is a self-learned and self-organized network without supervisory needs [7]. The CNN architecture contains three sorts of layers: convolutional, pooling, and fully connected. Also, contrary to other DL models, at least one layer of a CNN uses convolution operations. The regular layers of the CNN are explained in detail.

- Convolutional layers: A convolutional layer includes a collection of filters where the parameters need to be learned. The filter height and weight are smaller than the input. The filter is pushed over the input width and height, and the dot products between the input and the filter are measured at each spatial position in order to compute an activation map. Then, the convolutional layer output is computed by piling the activation maps of all filters. By downsampling the representation, the pooling layer decreases the number of parameters that need to be computed.
- Pooling layer: pooling operations produce single numbers of each region by taking small grid regions as input. Typically, either the max function (max-pooling) or the average function (average pooling) are utilized for computation.
- Dropout layer: during the training phase, dropout sets the outgoing edges of hidden units (neurons creating hidden layers) to 0 at random.

### 3.2. Fully Convolutional Network (FCN)

CNN architectures used patch-based approaches to classify the center pixel by analyzing a small patch of the whole image. Because of the large number of overlapping patches, these solutions needed an enormous amount of memory as well as exceptional computer power. The FCN architecture was proposed by Long et al. [8] to solve this problem using fully convolutional network architectures that can directly translate input images to ground truth pixel by pixel.

**Figure 1:**
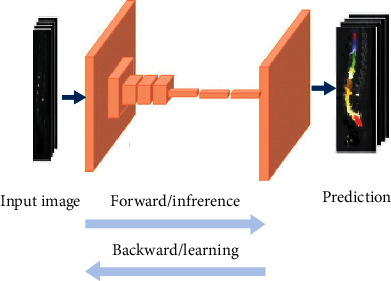
General architecture of the FCN.

In this way, the FCN greatly enhances the efficiency of training and the performance of models. The conventional FCNs used different scale bilinear upsampling methods to produce segmentation output with the same width and height as the input. These processes cause information loss and thereby reduce prediction accuracy. To enhance the performance FCN localization, high-resolution activation maps are combined with upsampled outputs and supplied to the convolution layers to gather the required output. The FCN has been the gold standard for image localization tasks since it was developed in 2015 [9]. The advanced FCN uses skip connections and creates U-Net replacing bilinear upsample operations of the FCN. An example of an FCN is shown in Figure 1.

### 3.3. U-Net Architecture

**Figure 2:**
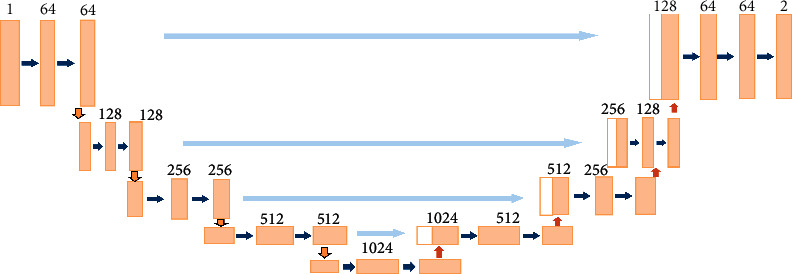
General architecture of U-Net.

In the realm of computer vision, semantic medical image analysis is of the utmost importance owing to its applicability in the supervised and texture feature extraction processes. U-Net and R-CNN are the two most widely used DL models in the analysis of medical images. Inspired by the promising performance of the FCN, Ronneberger et al. introduced U-Net [10], integrating spatial and deep semantics via skip connections and encoder-decoder pathways. Here, the encoder blocks use convolution layers to form contracting paths. Similarly, the decoder blocks form expanding paths with deconvolution layers. In U-Net, contracting paths are employed to collect context information while expanding paths to enhance localization precision. Furthermore, it uses skip connections between encoder-decoder pathways which look like U-shaped pathways. The structure of the U-Net network is shown in Figure 2.

### 3.4. Recurrent Neural Network (RNN)

In the RNN, the connections are recurrent in the network and enable to remember the previous input patterns. Consecutive slices are associated because the ROI is assumed to be distributed across many surrounding medical imaging slices (e.g., CT or MRI). The RNN captures data from the input slices in sequence. Since past and future input values can be used in a number of ways to modify the output value, new recurrent architectures have been proposed in the literature as an upgrade to the original RNN.

**Figure 3:**
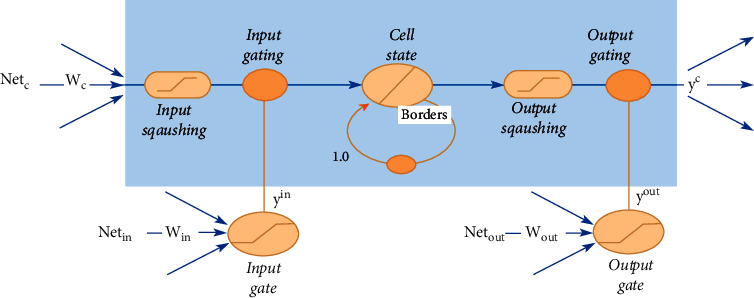
General architecture of LSTM.

The LSTM is the most common category of the RNN. In several ways, LSTMs provide advantages over traditional neural feedforward networks and RNNs. This is due to their ability to actively remember patterns over long time periods. The LSTM's chainlike architecture enables it to store knowledge for longer periods of time, thus addressing one of the RNN's challenges [11]. The LSTM integrates a memory unit that includes details about the inputs and is controlled by a variety of completely connected gates as shown in Figure 3. Therefore, the LSTM is able to extract localized spatiotemporal features. The three major parts of the LSTM are as follows:

- Forget gate: removes knowledge that is no longer required to complete the mission. This step is essential for the optimization of network performance.
- Input gate: responsible for loading cells with inputs.
- Output gate: sets the necessary information and outputs.

Since the LSTM has the ability to explore spatiotemporal features from videos, it has been considered for a number of applications to automatically localize ROIs in medical videos.

### 3.5. Challenges of Medical Image Analysis

Generally, it is a major challenge to analyze medical images visually. This is because these images suffer from inherent noise and low spatial resolution [12]. The variety of cell forms, edges, sizes, localized features, and organ locations has created a challenge for the efficient localization of objects during the analysis of medical images. One of the major obstacles in the localization of diagnostic images is the heterogeneous nature of the target organ. A target organ or lesion can differ enormously from patient to patient in scale and shape [13]. For example, the identification of vertebrae is quite difficult owing to the image artifacts generated by surgical implants, similarity in vertebrae appearance, and irregular pathological curvatures [13]. The difference in contrast between the ROI and the background needs to be taken into account when these images are used for automatic object localization. Furthermore, object localization needs to be matched with the template regardless of the displacement, rotation, size, or deformation of the object. Several methods are proposed in the literature for feature extraction that can aid in improving the precision of image localization. Traditional machine learning methods are built on handcrafted features which required a priori knowledge, and thereby these methods frequently failed to address these issues successfully.

Another challenge that can be faced when dealing with medical images is that obtaining the ground truth label images is difficult, especially when dealing with abnormal cases. This requires a substantial amount of technical expert knowledge to label images, and it is computation-intensive task. Each target object can be different according to tissue types and the shapes of organs. Furthermore, medical image localization usually involves 3D volumes. Analyzing 3D medical images can provide details of the human organ and can assist in detecting cancers, infections, abnormalities in organs, and traumatic injuries [14]. Therefore, more appropriate methods and techniques should be used for 3D volumes. However, for machine learning methods employed to deal with this type of data, a major issue that needs to be addressed is the efficiency of capturing high-dimensional parametric spaces. For instance, a 1024-dimensional input to the classifier is produced by a patch of 32 × 32 pixels. However, a 3D map of 32 × 32 × 32 has 32,768 voxels [15]. In contrast, 2D can increase the computation time as well the probability of overfitting, especially when the data are limited [16]. A vector with such a large input poses many problems, and the requirement for representative image features requires significant manual engineering effort.

Recently, robust and automatic medical image analysis approaches that can deal with these issues were in high demand to enhance the clinical process from diagnosis, the stratification of patients, treatment preparation, intervention, and follow-up [11, 17, 18]. Researchers are attempting to overcome these challenges in order to find the optimal methods for medical image analysis. For the past few decades, the implementation of DNNs to localize ROIs in medical images has been greatly increased. Also, it can be noticed from the literature that DNNs are widely applied for medical image analysis such as MRI [12], and traditional handcrafted features can be effectively replaced by the DNN. In DNNs, initial layers of filters are involved in extracting low-level image features and succeeding layers of filters are involved in learning higher-level image features.

Furthermore, DNN models offer a robust and efficient method to automatically locate ROIs in different types of medical images. DNNs can deal with the limitations of label datasets by applying weakly supervised learning. This implies that training the network for classification tasks achieves a localization task, and no extra labels are necessary. To solve the 3D issues, a DNN can be employed to automatically extract features from the raw images hierarchically. Later, these features are utilized in detecting the ROIs in 2D which can thereby enable to localize ROIs in 3D. This study will give an overview of using DNNs in medical image localization tasks.

## 4. Research Methodology

As stated earlier, the purpose of this systematic review is to discover and present literature on DL approaches developed for medical image localization via the formulation of research questions and the selection of relevant research articles. This study adopts the research methodology outlined by Kitchenham et al. [19]. The subsections that follow details review procedure, criteria for inclusion and exclusion, search and selection process proposed in the adopted methodology.

### 4.1. Review Process

Following the concept, guidelines, and measurements stated by Kitchenham et al. [19], we began our research with the establishment of a thorough review procedure. This procedure outlines the review context, search strategy, research objectives, and quality evaluation criteria for study selection and data analysis.

The review procedure is one that distinguishes a systematic review from a standard literature review. Also, it makes the review more consistent and less biased by the researchers. The reason for this is that researchers are required to disclose a search strategy and criteria for the inclusion or exclusion of existing studies in the review.

### 4.2. Data Collection Process

The method of data collection involved comprehensive research that presented DNN applications for localizing ROIs in medical images. These studies were studied to gather the appropriate information on the topic. The key purpose is to include a sample of the DNN applications. The three stages of the data collection process were as follows:

- Search phase: in this phase, articles were identified from reputable journals using keywords such as localization, DNN, CNN, and medical images.
- Selection phase: in this phase, articles were chosen and classified to meet the purpose of the survey. Then, the eligible articles were objectively analyzed.
- Review phase: in this phase, the chosen articles were reviewed, and the qualitative review results are described in our study.

### 4.3. Search Process

The search strategy adopted in our study involves both automated and manual search methods. The automated search method enabled the identification of primary research and helped to broaden the review viewpoint. Therein, we expanded the review including more new research on the review process. In accordance with Kitchenham et al.'s recommendations [19], we used the manual search method on the studies discovered by automated search to locate the study references.

In the automated search process, an extensive search for primary articles related to research title and keywords was conducted in virtual databases including IEEE Explore, ISI Web of Knowledge, Springer, and Elsevier to identify relevant research publications under the study. The literature from blogs, books, and magazines was not considered in the review process as the quality verification of those kinds of literature is not reliable. The keywords for the search process were derived from our study title and hypothesis. The search string used in the review process is as follows:

- (Medical image localization) and (deep learning or deep neural)
- (MRI or CT image) and (localization) and (deep learning or deep neural)
- (ultrasonic or ultrasound image) and (localization) and (deep learning or deep neural)

### 4.4. Selection Process

**Figure 4:**
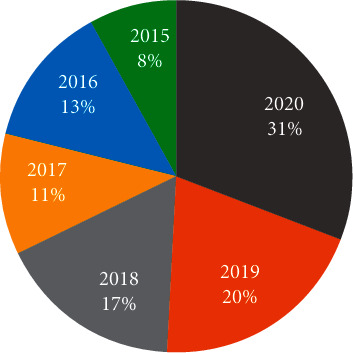
Trending chart depicting the temporal distribution of the chosen articles.

The selection was constrained to retrieve related studies published in the interval of the past five years from 2015 to 2020. In the search process, nearly 200 studies were extracted. Later, a manual screening process was conducted to select the most relevant literature. In the screening process, the duplicate articles, articles in which the application of the DNN for localization was not clearly described, and articles without full text were excluded. After exclusion, 94 articles were considered and accepted for further study. Table 1 and Figure 4 show the temporal distribution of these 94 articles against the past five years.

## 5. Evaluation of Reviewed Studies

There are varieties of applications of DNNs for localization tasks. This section addresses these applications and groups them based on the region that needs to be localized.

### 5.1. Vertebrae Localization

Degenerative disc disease is amongst the most frequent health problems that develop with age and causes lower back pain in adults. In this direction, the localization of the intervertebral disc (IVD) plays a crucial part in the detection of abnormalities [4]. Furthermore, with the recent development of technologies in the medical imaging domain, the emphasis on the automatic localization of IVDs is viewed as significant for the diagnosis of spine disease and for measurement quantification. Also, it is recognized as a crucial element for preoperative planning and postoperative evaluation of spine problems such as spinal stenosis, disc/vertebrae degeneration, scoliosis, and vertebral fractures [13, 20]. Identifying vertebrae is difficult because neighboring vertebrae generally share similar morphologies [11]. In reality, diseases frequently alter the anatomical structure of a vertebral column, making it difficult to distinguish the boundaries of the vertebrae [11].

In the past decade, several ML techniques are developed for automatic vertebrae localization in CT [11, 13, 21] and MRI volumes [20]. However, these methods fail to extract the diagnostic features that are most essential for accurate diagnosis of spinal disease. Recently, with the popularity of the DNN, researchers have employed DNNs to localize IVDs or vertebrae from volumetric data or 2D images [22–24]. The application of the DNN showed great promises with quantification measurement that is required for accurate diagnosis and early treatment of spine diseases. For example, the authors [21] employed a CNN on a public dataset of 224 arbitrary-field-of-view CT images taken from pathological cases. The researchers found that the CNN could detect vertebrae with 96% accuracy in less than 3 seconds. The authors determined that a CNN can deal with clinical diagnosis and therapy applications.

Another interesting approach to integrating image features from MRIs and CTs and automatically rectifying the position of vertebra is presented in [22]. The researchers designed the transformed deep convolution network (TDCN) for multimodal vertebra recognition. Feature learning is accomplished by using convolution-restricted Boltzmann machines (CRBMs). Then, the learned features can expose some unique microstructures in MR, CT, or MR/CT feature fusion. These feature maps are sent to two RBMs for multimodal feature fusion. These features and pose rectifications are naturally unified in a multilayer DNN. These methods are evaluated on MR and CT datasets (60 MRI includes T1-weighted and T2-weighted scans (T1 and T2 included) and 90 CT volumes) taken from different pathology cases (i.e., fractures and spondylolisthesis). The experiment results showed that the transferred CNN achieved 90% accuracy. This mode was able to provide a fully automatic location, name, and pose of each vertebra for routine clinical practice. The author declared that this new network could provide better promise for organ recognition with cross-modality images [22].

Automated localization of vertebrae in 3D spinal images has been addressed by several researchers in past literature. Chen et al. [13] presented CNNs to explore higher-level features from 3D CT images in order to automatically locate vertebrae. They designed a joint learning model with a CNN (J-CNN). This takes into consideration the appearance of vertebrae as well as the pairwise conditional dependency of neighboring vertebrae. Later, experiments were conducted to evaluate the proposed approach on MICCAI 2014 computational challenge data. They achieved smaller localization errors of 8.82 mm.

In [22], Cai et al. identified vertebra locations using a 3D deformable hierarchical model (DHM). The proposed model employs unsupervised learning to combine features extracted from many modalities and rectifies vertebral positions automatically. Also, the combination of features from CT and MR images enhances the discrimination ability of the feature representation and thereby boosts the vertebra patten invariance. This enables to analyze the images captured with varying protocols, resolutions, contrasts, orientations, and sizes. Generally, DNNs are capable of rectifying position and feature fusion. Therein, the proposed solution adopts unsupervised feature learning by stacking two layers of a convolution-restricted Boltzmann machine. The features extracted from these two layers are processed by stacking two restricted Boltzmann machines to achieve feature fusion. The system consists of two key modules. The local presence module in DHM extracts the cross-modality features and provides initial identification of the vertebra landmarks. The global geometry module in DHM uses point-based registration to match the spotted landmarks with the global spine model. The proposed system is able to identify locations, labels, and poses for local vertebrae. Furthermore, it can offer 3D spine reconstruction for desired spine sections or even the whole spine. This recognition method captured the local and global spine information from MRs and CTs. Chen et al. [13] employed a CNN with six layers to localize ROIs on 224 spine-focused CT scans. The intensity-based features of each selected voxel are passed to the CNN as an input. The CNN output represents the estimated relative distances between the voxel and the center of each vertebral body. These outputs are then transformed into absolute voxel locations in the image. The researchers found that the CNN can localize all visible vertebrae with an error of 18.2 in less than 3 seconds, whereas the random forest method achieved a mean error of 20.9 in more than minutes.

Jamaludin et al. [20] used a CNN to detect and localize multiple abnormalities in T2-weighted sagittal lumbar MRIs. The CNN model was trained to predict six different radiological scores and then generated six heat maps. Brighter hotspots in these heat maps were strong evidence for that region to affect the classification. The CNN was trained on only the “weak” supervision of class labels. It correctly localized pathology hotspots. The result demonstrated that the CNN can achieve good performance on multiple radiological scores, for example, disc narrowing, marrow changes, and endplate defects.

Motivated by a sequence in the vertebral order, Liao et al. [11] suggested a combination of an FCN and RNN to learn both the short-range and long-range contextual information. This method leverages the representational potential of the RNN while trying to integrate existing information about the spine scan. Such integration is essential because an FCN does not automatically know the morphology of an ROI, particularly when operating in 3D. This is because the computational complexity limits the scope of the network. A 3D FCN was used to effectively extract the short-range contextual features around the target vertebrae. LSTM is used to extract the long-range contextual features around the vertebrae of the visible spine column.

The idea is to use the FCN to convert a CT scan image into a sequence of spatially ordered vertebrae sample features. Then, the sequence features are passed to LSTM which has previously learned to encode the long-range contextual features among samples in the first stage. The SpineWeb dataset was used in this study [11]. There are 302 CT scans in this dataset. The authors claimed that their proposed approach outperformed previous studies by a significant margin with a 6.47 mean error. The experiment result demonstrated that the 3D FCN can encode the 3D spatial information of CT volumes to produce a more robust model than the 2D counterparts. Another interesting approach was published by Zheng et al. [25], who suggested a new approach to overcome this challenge by applying two steps. First, a simple network is applied for the initial testing of all voxels to capture a small number of optimal features. Then, a more accurate classification with a DNN is applied.

To increase the detection accuracy, deep learned image features are integrated with Haar wavelet features. This approach was tested on 455 patients to diagnose carotid artery bifurcation in a head-neck CT dataset. The authors showed promising results: they achieved a mean error of 2.64 mm. The authors suggested that the proposed method can be generalized to detect other 3D landmarks. Chen et al. [26] extended the 2D FCN into a 3D variant with end-to-end learning and inference, where voxelwise predictions were created. In their study, two different approaches were employed: one is a 2D FCN with deep feature representations by using adjacent slices, and the other is a 3D FCN with flexible 3D convolutional kernels. These two approaches were tested on the 3D MRI data of MICCAI 2015 challenge on automatic intervertebral disc localization and segmentation. The results proved that the 3D FCN obtained a better localization accuracy than the 2D FCN. Most of the previous studies dealt with localization as a classification task, and as such, generalized models of DL are leveraged. However, only a few approaches dealt with the localization of landmarks and regions directly from the image space [27]. For example, Payer et al. [28] used a CNN to directly detect landmark locations. They used a Gaussian to represent landmark maps as ground truth input data, and the CNN was straight trained to detect this landmark map. They evaluated different types of CNNs on 2D and 3D hand images. Each CNN was trained to find the presence of Gaussian heat spots centered at the landmark locations. In this study, the authors used the CNN with a large kernel and the CNN with downsampling techniques called (Downsampling-Net), and U-Net, as well as a novel SpatialConfiguration-Net. The last model was designed based on the integrated local appearance of landmarks with spatial landmark configurations that modeled anatomical variations.

The localization performance of the CNN was evaluated on two different datasets. The first one was 895 publicly available X-ray images of hands with 37 marked landmarks. The second dataset was 60 3D gradient-echo hand MR scans with 28 marked landmarks. The 2D CNN has six convolution layers, whereas Downsampling-Net has multiple blocks containing two convolution layers followed by pooling layers. After the last block, two additional convolution layers are joined. The U-Net consists of a contracting path similar to Downsampling-Net, and an increasing path composed of upsampling blocks, concatenation with the same level of output from the contracting path, and last, two convolution layers. The 2D SpatialConfiguration-Net has three convolution layers followed by a spatial configuration block. The experimental result demonstrated that heatmap regression based on CNNs obtained a good localization performance for both 3D and 2D datasets. In addition, SpatialConfiguration-Net was robust on limited amounts of training data. The previous study showed a reasonable method for hand X-ray images, although the authors [29] declared that this method could not adapt well to body parts and scan protocols in which the area of orientation, size, and acquisition differs. Yang et al. [29] developed the deep image-to-image network (DI2IN). In DI2IN, the function layer concatenation is similar to 3D U-Net. The shortcut bridges to decode layers are set up directly from the encoder layers. The DI2IN was trained on 1000+ 3D CT volumes from different patients. The result outperformed other state-of-the-art methods with a 90% localization accuracy.

Multimodality MR images for the same subject can be attained using various scanning configurations. This shows valuable and comprehensive information which can help to provide more robust diagnoses and treatment. DNNs have been taken into consideration to analyze this type of medical image. For example, in [9], an FCN was used on multimodality 3D MR data. The authors aimed to develop a novel multiscale and modality dropout learning model to locate IVDs from four-modality MR images. The first step was to design a 3D multiscale context FCN in order to learn high-level features. These features can improve the representation capability of the network to deal with the scale variation of anatomical structures. Second, to accompany the information from different modalities, a random modality voxel dropout strategy was used to enhance the performance of IVD localization. The approaches were tested on 24 sets of 3D multimodality MR scans acquired from 12 patients. The dataset was obtained from the 2016 MICCAI Challenge on Automatic Localization and Segmentation of IVDs from Multimodality MR Images 2. The experiment outperformed the MICCAI-2016 challenge with a dice score of 91.2% [9].  Table 2 provides the list of various studies illustrating the efficiency of the DNN for the analysis and the localization of abnormalities in medical images.

### 5.2. Anatomical Plane Localization in Foetal Ultrasound (US)

The automated localization of the anatomical planes in foetal US images is another critical clinical problem to be addressed. This is attributed to a variety of factors such as noise and the small size of the foetus images. Recent methods used for plane localization focused on only detecting planes within specific body regions. In recent years, DNNs are also utilized for the localization of scan planes and important frames of US images. For example, Ghesu et al. [76] developed an adaptive DNN powered by marginal space DL for aortic valve localization in US images. The proposed model is able to detect nonrigid object boundaries. A dataset consisting of nearly 2891 volumes of images collected from 869 patients is employed to test and validate the correctness of the model for aortic valve localization in 3D US images. The mean error achieved in this study was 1.83 mm. Another study [77] focused on using a transferred CNN (T-CNN) to represent high-level features appearing on the foetal abdominal standard plane in US images. US video was obtained from a sweep on pregnant women (between 18 and 40 weeks of gestational age). Every video was obtained from one patient and contained between 17 and 48 frames. Three US standard planes, namely, abdominal, face axial, and heart four-chamber view were used to collect training set with approx. 45000 US images. The T-CNN outperformed the state-of-the-art method for foetal abdominal standard plane (FASP) localization with 89.6% accuracy.

The authors [78] extended the work of Ghesu et al. by developing a knowledge-transferred RNN (T-RNN) to localize the foetal standard plane. The T-RNN consists of a CNN and LSTM model. The researchers attempted to combine a deep hierarchical spatiotemporal feature extractor learning model. Detection was first implemented by using a joint-learning CNN to locate ROIs in US images. Then, LSTM was used to explore the temporal features based on the ROI features in two successive frames extracted using the CNN model. At last, the score for each frame was computed by taking the average of all LSTM model predictions. Then, the frame with a score higher than a determined threshold is detected as a standard plane. The proposed approach was compared with the T-CNN. The effectiveness of the integrated T-RNN was demonstrated by the results obtained from three US standard planes. The T-RNN showed better accuracy with 0.908, 0.867, and 0.867 on the three standard planes, respectively. The T-RNN showed an ability to explore spatiotemporal features for standard plane localization in US images.

Baumgartner et al. [79] also employed the CNN model for standard plane localization in US video frame data. Besides, CNN performance was evaluated on 1003 midpregnancy scans. The experimental results showed the CNN potential to detect standard planes with a recall and precision of 80% and 69%, respectively. The researchers concluded that the CNN allows for real-time inference with precise localization for anatomical planes in foetal US images.

Another work aimed to address the challenges of US foetal imaging by emphasizing the key features in US images. Kumar, et al. [80] designed a new general approach for localization based on training two CNNs to learn the saliency features. These learned features can discriminate different planes in US images. The best result achieved was 93.7% for the head, 82.6% for the femur, and 71.6% for the spine. Another article [81] introduced a new CNN based on a VGG-16 model called sonography (SonoNet). SonoNet employed weak supervision to learn from labeled images only and localize 13 standard scan planes. The SonoNet consisted of 3 fully connected layers and 13 convolutional layers. The study evaluated 2694 2D US images taken from gestational women between the ages of 18 and 22 weeks. A training set with 50 images that were collected from each of the 13 standard scan planes was used to train SonoNet. These images were used as ground truth after they were labeled manually with bounding boxes. The intersection over union (IOU) metric was computed to find the similarity of the automatically determined bounding box to the ground truth. The result demonstrated an accuracy of 77.8%. The key points of the above discussed literature that use the DNN for anatomical plane localization in foetal ultrasound are given in Table 3.

### 5.3. Anatomical Structure Localization in MRI

In noninvasive neuroanatomical investigations, segmentation of the whole brain on a structural MRI is crucial. In [82], the authors presented a new method called Spatially Localized Atlas Network Tiles (SLANT) leveraging the advantage of canonical image processing techniques and deep learning approaches for segmenting the whole brain. The proposed method distributed multiple independent sets of 3D networks to cover the overlapping subspaces in the Atlas space. Furthermore, the authors constructed auxiliary labels from 5111 unlabeled scans to enhance the learning performance of the proposed SLANT method on 133 labels while using just 45 manually labeled training data. In three validation cohorts, the proposed method achieved 0.78, 0.73, and 0.71. Furthermore, the proposed method lowered the computing time from more than 30 hours to 15 minutes. In [83], an FCN-based model called VP-Nets was used to show a new, more efficient way to detect multiple brain structures in 3D foetal neurosonography. The model required limited training data and was learned from weakly labeled volumetric images. Its use is readily transferable to different medical imaging modalities if detection is desired. The prediction accuracy of models with different depth and feature channels was reported. The findings showed that the proposed model generalized effectively across the dataset. Also, the visualization of the 3D saliency volume revealed the potential of the proposed solution in locating essential structures in 3D space without the need for supervision.

### 5.4. Landmarks Localization in Medical Image

Another interesting implementation of the DNN was to localize landmarks on the distal femur surface. Yang et al. [17] used CNNs to identify these landmarks. Here, three sets of 2D MRIs acquired along three respective planes were processed using CNNs. The intersection of the three 2D slices with the highest classification output defined the location of ROIs in 3D. Another study by Kong et al. [84] used the frames of cine-MRI of the heart to identify the end-systole and end-diastole. Here, the researchers developed a temporal regression network named TempReg-Net. The proposed network combined an LSTM with a CNN where the LSTM was employed to translate the temporal features and the CNN was employed on cardiac sequences to encode the spatial features. In addition, TempReg-Net was trained to introduce a new loss function to enhance the prediction accuracy of the label structures. The network demonstrated a mean difference of only 0.4 frames when validated on cardiac sequence datasets. Also, another piece of literature published in 2015 [12] presented a new method based on the CNN for left ventricle localization of cardiac MRI. Here, the CNN performed feature extraction with six layers comprising kernels of different sizes. A database of 33 patients was used in this study. The research findings proved the potential of the proposed CNN model in terms of sensitivity, specificity, and accuracy achieving 83.91%, 99.07%, and 98.66%, respectively.

Another study [85] developed a landmark detection system by using an FCN. The aim of this system is to locate 22 landmarks in 3D head CT scans. The system is based on two models. The first model is based on an FCN trained only on images, and the model was pipelined with the second model, where these inputs provide spatial information. The second model is trained from images as well as spatial features that are provided by the first model. This integration of learned features is called the Atlas location auto-context. The FCN is built with six layers. The authors claim that the performance of the FCN was better than that of the decision forest methods. Furthermore, they declared that the FCNs showed precision that is nearly similar to that of a human observe [85].

Other approaches were developed by combining numbers of CNNs. For example, Bob et al. [86] developed a DNN model combining three CNNs for ROI localization. Here, the proposed model was employed on anatomical regions such as descending aorta, aortic arch, and heart. Furthermore, each CNN in the proposed model was employed to automatically analyze one orthogonal image plane and localize the ROI in that plane. Later, the output was combined to provide a 3D bounding box around it. The performance of ROI localization was evaluated by computing median dice scores. The result of this experiment showed that the average dice measure in determining bounding boxes around the ROI, namely, aortic arch, descending aorta, and heart were 0.70, 0.85, and 0.89, respectively. These achievements proved the potential of the CNN for the 3D localization of anatomical structures [86].

Yan et al. [87] focused on designing a two-stage DL model to identify a body part where the first stage of learning involved discovering the local regions and the second stage of learning aimed at learning the identifiers from the discovered local regions for discriminating the body parts. During the pretraining phase, the proposed CNN adopted multi-instance learning for extracting the most discriminating identifiers from the training slices. Later, the pretrained CNN is further boosted in boosting phase using the discovered local regions. Thus, the authors claimed that the proposed solution outperforms the global image-based context models. Besides, the proposed model leveraged the benefits of multi-instance learning and was capable of automatically discovering the local region without the need for manual annotations of local regions in the provided training set. The proposed solution outperforms the state-of-the-art approaches on both synthetic and whole body datasets.

A reliable organ localization method was presented by Xiaoguang et al. in [88] with substantial anatomical and contextual variability from 3D volumes. The search space in 3D spatial is divided into two parts: slice and pixel. Both of these parts are modeled in 2D space. Different learning architectures were used for each component to leverage the respective modeling power to three orthogonal orientations in a global and local context. In learning-based localization methods, slice scanning along each orientation is used instead of the usual patch-based scanning. This greatly minimized the number of model assessments. The target organ location is determined by combining data from three orientations and learning architectures. In this experiment, 499 CT scans were used to evaluate this method. The result showed the robustness and promise of the presented method.

Using a CNN, De Vos et al. developed an automated localization method in [89] for one or more anatomical structures in 3D medical images. This is done by recognizing their existence in 2D image slices. Furthermore, a single CNN is employed in the proposed solution to learn and identify the anatomical structures in 3D medical images. Spatial pyramid pooling is used to enable the CNN to evaluate slices of varying sizes. All slices of the CNN are combined to form 3D bounding boxes after detection. The experiment study employed abdominal CT, cardiac CT angiography, and chest CT images. Here, chest CT identified aortic, ascending, and descending arch, and heart whereas abdominal CT scans identified the liver and cardiac CT scan identified the left ventricle. Here, the difference between the centers and edges of reference bounding boxes that were set up automatically and by hand were used to assess localization. The proposed solution produced best results when the structural border was well defined (e.g. aortic arch) and worst results when the border was not well defined (e.g. liver). Thus, the proposed solution delivered more accurate and robust results in locating multiple structures.

The automated localization of pulmonary nodules on CT scans was also investigated based on DNNs. In [90], an automated approach for pulmonary nodule localization on lung CT is presented. The approach segmented voxel-level nodule accurately despite it involving weak supervised learning and just image-level labels as training data. Furthermore, the proposed solution modifies the image classification CNN to learn discriminative regions from feature maps of convolutional units at various scales, and a unique candidate-screening approach is presented to identify the real nodule location. The proposed weak supervised DNN for pulmonary nodules localization outperforms a fully supervised CNN-based algorithm on the public LIDC-IDRI dataset.

Another interesting application of the CNN was to detect coronary artery calcium (CAC) score in coronary CT angiography (CCTA) [91]. This study included CCTA images of 50 patients, and these images were split evenly among the five groups with the highest risk of heart disease. The CNN was trained to identify CAC voxels. The learned features extracted from the CNN were classified using a Random Forest. The volume of the CAC was quantified for each patient and then compared to the manual annotations of CCTA image. The results showed that the CNN was able to automatically detect CACs and was quantified in CCTA. According to the researchers, this strategy may eliminate the requirement for a CT scan for CAC scoring, which is usually performed prior to the CCTA scan. Thereby, the proposed solution is expected to lower the radiation dosage given to the patient during CT. Although the localization of weakly supervised objects is technically useful because they avoid the need for fine-grained annotations, especially in the domain of medical imaging, the unavailability of priors may have difficulty dealing with this type of learning.

In [92], the authors designed a weak supervised self-transfer learning (STL) framework in order to localize a lesion. Both classification and localization networks were integrated on STL networks to enhance the localization performance by focusing on the correct lesions without any types of priors. Experimental results on chest X-rays and mammogram datasets demonstrated that The STL framework has significantly better localization than previous weakly supervised localization approaches. Table 4 provides a literature review on the application of the DNN for landmark localization.

## 6. Key Findings and Recommendations

The most challenging problem that most researchers could face when dealing with medical images is the limitation of the dataset. The recent achievements and successes of DNNs are due to their training on large datasets such as ImageNet. Here, the appearance of these images varies greatly in terms of color and intensity. Also, these images vary in characteristics as they are captured at different angles and distances. On the contrary, medical images are ambiguous in nature and characteristics. Consequently, variation is less compared to the typical image datasets. Besides, DNNs are not efficient in learning when the training samples are limited. Therefore, there is an essential requirement to train DNNs on relevant feature representations of medical images which require enormous training samples. This is considered the most challenging problem that most researchers could face when dealing with medical images. In this way, medical images can be used to fine-tune CNN models that have already been trained on datasets of natural images. This method, called transfer learning, has been used in a wide range of medical imaging applications with great success. Even though DNNs require extensive computational resources for their successful implementation, 3D DNNs have excelled in several fields with outstanding performance. Also, training 3D DNNs demands a large number of parameters, and this situation is exacerbated further in 3D medical images and restricts medical image resizing without considerable loss of information. This area of research is still in its infancy.

In DL, feature representation learning is a tedious and complicated task as it is very difficult to ensure whether the model is effective in capturing the most discriminative features from the given datasets that are required for the successful accomplishment of subsequent tasks. DNNs have demonstrated their promising performance in handling raw images without the requirement for manual preprocessing and feature extraction process. DNNs are superior in identifying and learning the discriminating features from the image data. Although CNNs have enabled feature encoding in latent space to be much easier, it is highly crucial to evaluate if other variants of the DNN are capable of learning features that are generalizable across datasets as highlighted in [14]. Finally, the ultimate challenge is the unavailability of labeled image datasets, the high expense required in labeling the datasets, and the absence of consensus among the experts over the given labels. However, this challenge can be addressed by leveraging the advantage of data augmentation techniques to generate examples with known ground truths.

## 7. Conclusion and Future Directions

Different implementations of DNNs were addressed by various publications. Localization with DNNs tends to be the most common overall technique for recognizing organs, regions, and landmarks with acceptable results. Many studies used DNNs by adapting weak learning to enhance localization performance. Other studies focused on combinations of features such as spatial and temporal features by using two types of DNNs (CNN and RNN). RNNs have demonstrated potential in localization performance in the temporal domain. The combination of different feature domains enhances the discrimination of feature representation and allows to process the images automatically with different contrasts, resolutions, protocols, and even different sizes and orientations.

Despite the application of the DNN in the field of medical image localization has shown tremendous progress, these methods are still inferior to the performance experts giving room for further improvements. Hence, future research can focus on employing some notable DL structures such as quantum learning, ensemble learning, and U-Net. Also, the researchers can enhance the state-of the art DNN methods with advanced pretraining and training strategies. Apart from that, the performance of state-of-the-art DNN methods can be investigated against the class imbalance problems and efforts can be put forward to develop techniques that can achieve performance gain under class imbalance data.

The key limitation of this review is that it presents only the most popular DNN architectures and their applications for medical image localization. Furthermore, attributed to the enormous corpus of active research in medical image analysis, this review does not include all variants of the DNN. Almost the majority of the DNN architectures reviewed in this study are supervised models. Due to the limited allowed margin of error, supervised DNN approaches are involved in most of the proposed medical image localization tasks. Unsupervised DNN architecture is uncommon in biomedical imaging, although research is underway.

Summing up, this review work provides an insight into designing an efficient DNN structure for medical image localization tasks and also puts forward a few directions for future researchers to leverage fully the potential of the DNN for accurate localization tasks.

## Figures and Tables

**Table 1:** Temporal distribution of primary articles accepted for the study.

Year	Number of articles
2020	29
2019	19
2018	16
2017	10
2016	12
2015	8

**Table 2:** State-of-the-art literature on the DNN for localization of abnormalities.

Reference	Adopted DNN	Dataset used	Research findings
Onthoni et al. [30]	Pretrained CNN	10,078 CT images from autosomal dominant polycystic kidney disease	mAP = 94% localization of polycystic kidney
Roy et al. [31]	STN	Italian COVID-19 lung ultrasound database (ICLUS-DB), containing 277 LUS videos from 35 patients	F1-score of 65.1 for localizing COVID-19 lesion on LUS images
X. Wang et al. [32]	U-Net	630 CT scans	Accuracy of 0.901 for localizing of the COVID-19 lesions
Noothout et al. [33]	FCN	Two datasets, one containing 61 olfactory MR scans and another consisting of 400 cephalometric X-rays from ISBI 2015 grand challenge images	Distance errors were 1.45 mm for the the right and 1.55 mm for the lift in the localization of the coronary ostium
Cano-Espinosa et al. [34]	U-Net	CT images	DICE coefficients of 0.875, 0.914 for PMA and SFA, respectively, in CT images
W. Jiang et al. [35]	ResU-Net	Dataset containing 60 cases of MR images from 30 patients available for MICCAI WMH segmentation challenge	DICE coefficient of 0.832 for localizing WMH
Zhou et al. [36]	CNN	MRI images taken from 1537 patients	Dice distance of 0.501 ± 0.274 for breast cancer localization
Lin et al. [37]	U-Net	MRI (169 patients with cervical cancer stage IB–IVA captured; diffusion-weighted (DW) images)	Positive predicted value 0.92 for cervical tumors localization in (MRI)
Trebeschi et al. [38]	CNN	140 patients with biopsy proven locally advanced rectal carcinoma (LARC)	Dice Coefficient 0.68 for localization of rectal cancer in MRI
Liang et al. [39]	CNN	FFDM images for 779 positive cases and 3018 negative cases	Positive 0.52 localization of breast cancer
Naseer Bajwa et al. [40]	CNN	Online retinal fundus image database for glaucoma analysis and research. High-resolution fundus (HRF) image database optical coherence tomography (OCT) and color fundus images of both the eyes of 50 healthy persons	Accuracy 100% for localized optic disc
Reena and Ameer [41]	AlexNet	Microscopic blood images (257 cells belonging to five types of leukocytes)	Mean average precision 98.42% for leukocytes localization
Fan et al. [42]	CNN	Four datasets, namely, BCSI, LISC, and two other medical datasets	Dataset 1 0.99544, Dataset 2 0.99432, BCSI 0.98947, and LISC 0.98443
Huang et al. [43]	VGG-16	Diffusion kurtosis images of 59 patients with epilepsy lesions in the hippocampus	Accuracy 90% for localization of epileptic foci
Heutink et al. [44]	CNNs	123 temporal bone CT scans were acquired with two UHR-CT scanners	Error 8% for localization
Cheng et al. [45]	CNN	PXR dataset (frontal pelvic radiograph)	Accuracy of 95.9% for localization of hip fractures on plain frontal pelvic radiographs
González-Gonzalo et al. [46]	VGG-16	The kaggle DR dataset with 35,126 images from 17,563 patients	False positive 0.71 for localization of diabetic retinopathy (DR) and age-related macular degeneration abnormalities
Mwikirize et al. [47]	CNN	2D B-mode US images	Localization of needles inserted both in-plane and out-of-plane US image
Man et al. [48]	U-Net	NIH dataset with 82 contrast-enhanced abdominal CT images	Recall rate 0.9 for pancreas localization on CT images
Y. Q. Jiang et al. [49]	GoogleNet	8046 microscopic ocular images	Mean intersection over union 0.863 for localizing basal cell carcinoma
Roggen et al. [50]	Mask R-CNN	X-ray images from 12 abdominal cancer patients	
Shen et al. [51]	CNN	NYU breast cancer screening dataset	Accuracy 78.1% for localized malignant lesions
Joel et al. [52]	CNN	637 cone beam CT volumes	The mean curve distance 0.56 mm for localization of the mandibular canals
Winkler et al [53]	CNN	Six dermoscopic image sets. Each set included 30 melanomas and 100 benign lesions	Accuracy 93.3% for melanoma localization
H. Wang et al. 2020 [54]	CheXLocNet	SIIM-ACR pneumothorax segmentation dataset 2079 radiographs	Dice score of 0.72 for localized pneumothorax lesions in chest radiographs
Poon et al. [55]	DL	291,090 colonoscopy videos	Polyp-based sensitivity=96.9 %
Urban et al. [56]	CNN	8,641 images from screening colonoscopies collected from 2000 patients	Accuracy, 95% for polyp-localization
Ouyang et al.	DL	CXR (NIH ChestX-ray14 and CheXpert)	
Guan et al. [58]	CNNs	ChestX-ray14 collects 112,120 frontal-view images of 30,805 patients [59]	AUC 0.871 for localizing pneumonia infection in CXR
Rajaraman et al. [60]	CNNs	Radiological Society of North America (RSNA) CXR dataset [61]	mAP 0.317 for localizing abnormalities on CXR
Rajaraman [62]	VGG-16	CXR [63]	Accuracy 93.6% for localizing pneumonia infection in CXRs
Kermani et al. [64]	NF-R-CNN	3250 axial CMR images for 65 patients with ARVD	Mean error 7.33 ± 8.1 for heart localization in cardiac MR images
Vaiyapuri et al. [7]	DL	The dataset holds a total of 500 CT images, with 250 images of pancreatic tumor and 250 images of nonpancreatic tumor	Near-optimal ACC of 0.9840, and a max ACC of 0.9935 on CT images towards pancreatic tumor localization
Groves et al. [65]	CNN	3825 US images	RMSE of 0.62 and 0.74 mm in the axial and lateral, respectively
Xue et al. [18]	CNN	IHC images of colon tissue	Accuracy, 92.69% for protein subcellular localization
Al Arif et al. [66]	FCN	296 lateral cervical spine X-ray images	Dice similarity coefficient of 0.94 for spine localization
Won et al. [67]	CNN	MR images	Accuracy 77.5% for localizing the center position of the spine canal
Peña-Solórzano et al. [68]	CNN	3D whole body CT scans	Dice scores of 0.99, 0.96, and 0.98 were obtained in the axial, coronal, and sagittal views for femur localization
Goyal et al. [69]	CNN	1775 images of DFU	Mean average precision of 91.8% for diabetic foot ulcers localization (DFU)
Afshari et al [70]	CNN	479 imaging captures the metabolic activity of tissue (PET scans) taken from 156 patients [71]	Localization error 14 mm for localized anatomical objects in PET scans
Sarikaya et al [72]	CNN	Video data of ten surgeons performing six different surgical tasks	Precision of 91% for localization in robot-assisted surgery RAS videos
Davidson et al. [73]	CNN	290 images of 142 healthy retinas and 148 retinas afflicted by Stargardt disease, acquired from 8 subjects with Stargardt disease	Dice score of 0.9577 for cone localization in images of healthy retinas
Dolz et al. [4]	U-Net	IVD dataset is 16 3D multimodal MRI images	Localization error was 0.4
Arik et al. [74]	CNN	Cephalometric X-ray image dataset which includes 19 anatomical landmarks	75.58%, 75.37%, and 67.68% accuracy for localizing sella, gonion and articulate landmarks
van der putten et al. [75]	CNN	494,355 endoscopic images	Accuracy 92%

**Table 3:** State-of-the-art literature on the DNN for localization of anatomical structure.

Reference	Adopted DNN	Dataset used	Research findings
Baumgartner et al. [79]	CNN	US video frame data with 1003 midpregnancy scans	Precision, 69% and recall, 80%,
Ghesu et al. [15]	Adaptive DNN with marginal space learning	3D US images collected from 869 patients with 2891 volumes	Accuracy, 45.2%
Chen et al. [77]	CNN with transfer learning	11,942 foetal abdominal US images collected from pregnant women	Accuracy, 89.6%
Chen et al. [26]	Extended (Chen et al., 2015b) with LSTM to extract spatial temporal features	11,942 foetal abdominal US images collected from pregnant women	Accuracy, 90.8%
Baumgartner et al. [81]	CNN based on a VGG-16 model	2694 2D US images taken from gestational women	Accuracy, 77.8%

**Table 4:** State-of-the-art literature on application of the DNN for landmark localization.

Reference	Adopted DNN	Dataset used	Research findings
Yang et al. [17]	CNN with shape statistics	Three sets of 2D MRI slices	Minimum error rate of 1.61% for landmark localization
Kong et al. [84]	LSTM with a CNN with new loss function	MRI cardiac sequences collected from 420 patients	Average frame difference (aFD), 38%
Emad et al. [12]	CNN with different kernel sizes	MRI cardiac sequences collected from 33 patients	Accuracy, 98.66%
de vos et al. [86]	Fusion model with three CNNs	Dataset containing 100 low-dose CT images	Median dice score of 89% for heart
Yan et al. [87]	CNN with multistage and multi-instances learning	Two datasets: first with synthetic dataset and later with whole body CT scan dataset	Accuracy, 89.8%
Lu et al. [88]	Different DL architectures considering the orthogonal orientations	499 patient CT body scans	Error rate < 2.0 on average for organ localization
de vos et al. [89]	CNN with spatial pyramid pooling	Three different datasets with 200 chest CT, 100 cardiac CT angiography (CTA), and 100 abdomen CT scans, respectively	F1-score > 95% for all three datasets
Feng et al. [90]	CNN extracts features at different scales for voxel-level nodule segmentation	Public LIDC-IDRI dataset	Accuracy, 88.4%
Wolterink et al. [91]	Leverages CNN for feature extraction and Random Forest for coronary artery calcification	Cardiac CT angiography (CCTA) from 50 patients	Accuracy, 0.8
Hwang and kim [92]	STL with joint optimization of classification and localization simultaneously	Two datasets, namely, CXR and mammogram	Accuracy, 83.69

## Data Availability

Data sharing not applicable to this article as no datasets were generated during the current study.
